# Sustaining Progress towards NTD Elimination: An Opportunity to Leverage Lymphatic Filariasis Elimination Programs to Interrupt Transmission of Soil-Transmitted Helminths

**DOI:** 10.1371/journal.pntd.0004737

**Published:** 2016-07-14

**Authors:** Arianna Rubin Means, Kristjana Ásbjörnsdóttir, Charles Mwandawiro, David Rollinson, Julie Jacobson, Tim Littlewood, Judd L. Walson

**Affiliations:** 1 Department of Global Health, University of Washington, Seattle, Washington, United States of America; 2 The DeWorm3 Project, The Natural History Museum of London, London, United Kingdom; 3 Eastern and Southern Africa Centre of International Parasite Control, Kenya Medical Research Institute, Nairobi, Kenya; 4 Neglected Tropical Disease Team, Bill & Melinda Gates Foundation, Seattle, Washington, United States of America; 5 Departments of Global Health, Medicine, Pediatrics, and Epidemiology, University of Washington, Seattle, Washington, United States of America; Centers for Disease Control and Prevention, UNITED STATES

In light of the unprecedented momentum to control or eliminate ten neglected tropical diseases (NTDs) by 2020, the NTD community is at a crossroads of opportunity. Efforts to eliminate lymphatic filariasis (LF) using mass drug administration (MDA) platforms have dramatically and simultaneously increased the number of individuals treated for soil-transmitted helminths (STHs) in many areas, owing to overlaps in endemicity and drug efficacy. A concomitant reduction in STH infection raises the possibility of moving beyond current World Health Organization (WHO) morbidity control guidelines to actual disease elimination for STHs [1]. We identify specific rationale, opportunities, and challenges associated with leveraging existing LF elimination platforms for the purpose of interrupting STH transmission.

Over 1,500,000,000 people globally are infected with STHs, including roundworm (*Ascaris lumbricoides*), whipworm (*Trichuris trichiura*), and hookworm species (*Necator americanus* and *Ancylostoma duodenale*). STHs are transmitted when infected persons shed eggs in feces that then contaminate soil or water and are ingested by or penetrate the skin of others. An individual’s worm burden, or the intensity of their infection, is directly related to their risk of STH-associated morbidity. Moderate to heavy STH infections are associated with diarrhea, malnutrition, chronic malaise, impaired cognitive development, disrupted linear growth, chronic inflammation, and anemia [2].

Current WHO recommendations focus on the control of STH-associated morbidity via MDA of albendazole (ALB) or mebendazole, often donated to endemic countries by the pharmaceutical industry. Target populations include pre-school age children (PSAC), school age children (SAC), and women of childbearing age, including pregnant women in their second and third trimesters. The WHO strategy for SAC recommends that MDA programs target SAC annually where prevalence is 20%–50% and biannually where prevalence is over 50%. These programs are primarily delivered using school-based platforms with teachers serving as drug distributors. In alignment with the WHO NTD Roadmap, this sustained control strategy aims to decrease worm burden and reduce STH-associated morbidity by ensuring that 100% of endemic countries are reaching PSAC and SAC with at least 75% coverage by 2020 [3].

However, in 2013, only 39% of SAC and 49% of PSAC in need of treatment were reached with MDA, and only 27% of endemic countries currently attain London Declaration coverage targets [4]. As endemic countries aim to rapidly scale up STH programs to achieve the 2020 goals, they are also faced with the challenge of sustaining programs indefinitely to continue morbidity suppression in vulnerable populations. This is largely because most STH strategies do not address adult reservoirs of infection; in some cases, up to 80% of hookworm parasites and 30% of *Ascaris* and *Trichuris* worms are harbored in adult populations [5–7]. Recent evidence from mathematical models suggests that in some settings, interrupting STH transmission using MDA may only be feasible if annual treatment is broadened to include adults [8].

A potential strategy for broadening the reach of STH programs is to leverage the existing community-based infrastructure of LF elimination programs. LF (including *Wuchereria bancrofti*, *Brugia malayi*, and *Brugia timori* species) is a disease caused by mosquito-transmitted parasitic worms that damage the human lymph system, resulting, most recognizably, in lymphedema, elephantiasis, and hydrocele. The WHO strategy for LF recommends annual MDA of ALB and either ivermectin or diethylcarbamazine (DEC) to all individuals living in endemic areas beginning at two years of age (for the ALB and DEC regimen) or five years of age (for ALB and ivermectin). Following four to six annual rounds of MDA, the density of circulating microfilaria in infected individuals (and thus the community-level force of infection) may be reduced and LF transmission disrupted.

Over 1,000,000,000 people across 55 countries are in need of MDA for LF [9]. Despite this large target population, LF elimination programs have demonstrated success in interrupting disease transmission of the infecting organism in many settings. Transmission assessment surveys (TAS) determine whether a given implementation unit meets specific operational and infection criteria and can successfully stop MDA for LF and transition to post-MDA surveillance. To date, 46 countries have implemented a TAS to assess readiness for cessation of MDA in at least one implementation unit, and 18 national LF programs have successfully moved to post-MDA surveillance [9].

There are a number of compatibilities between LF and STHs that would facilitate a coordinated LF surveillance and STH disease elimination strategy. First, the diseases are geographically congruent; 72 countries are co-endemic for LF and STHs, and in 2014, 85% of the population in need of treatment for STHs lived in LF-endemic countries [10]. Second, both implementation of the LF TAS and routine STH prevalence surveys require sample collection from school-age populations, although sample types differ. Third, both LF and STH programs utilize MDA with ALB as chemotherapeutic interventions, and ivermectin (used in areas co-endemic for LF and onchocerciasis) also has efficacy against STH infections, including *Trichuris* [11].

As a result, community-based LF programs have undoubtedly already contributed to the decline of STHs in co-endemic areas. After two rounds of LF MDA with DEC and ALB in Haiti, the prevalence of *Ascaris*, *Trichuris*, and hookworm was reduced by 24.9%, 55.3%, and 82.1%, respectively. Significant reductions in infection intensity were also observed. Additionally, although women of reproductive age were not provided ALB during LF MDA, *Trichuris* infection prevalence decreased comparably across women, children, and men, indicating that LF MDA may have reduced the force of STH infection [12]. Similar reductions in STHs were observed amongst SAC in Tanzania following MDA with ivermectin and ALB for LF [13].

Transitioning community LF MDA programs to STH programs that deliver treatments to PSAC, SAC, and adults may reduce residual infections harbored in non-SAC populations as well as in SAC who are not enrolled in school, and thus be a viable approach to sustaining progress toward interrupting STH transmission. The acceptability associated with many LF programs would benefit STH programs as they engage with new target populations. Finally, although STH resistance to benzimidazole anthelmintics has not been confirmed in humans, it has been observed in nematode infections of livestock [14]. One theoretical approach to decreasing the risk of inducing resistance is to shorten the duration of drug pressure by broadening target populations and more rapidly interrupting STH transmission.

For these reasons, leveraging the existing infrastructure (including the health workers, supply chains, and delivery schedule) and success of LF programs may be a unique opportunity to facilitate a smooth transition to a community-based STH platform. At the same time, LF programs that have achieved post-MDA surveillance status will continue to benefit from an ongoing infrastructure to facilitate the surveillance activities necessary for monitoring potential recrudescence or collecting data for WHO elimination dossiers. There are a number of potential challenges to integrating LF and STH platforms. Most notably, utilizing community-based LF platforms to serve as the primary method to deliver STH services will require political as well as logistical changes. Where STH programs are primarily administered by ministries of education, there will need to be close coordination with ministries of health. Methodological innovations will also need to accompany the proposed programmatic shift to ensure that coordinated sampling methodologies are validated and field deployable diagnostics are available for relevant coordinated surveys. This presents a major challenge that will require technologies and processes to enable the simultaneous sampling, storage, and testing of fecal and serum specimens.

The design of relevant field studies will need to account for complex issues such as randomization of communities on various LF elimination trajectories and with varying access to co-interventions such as water and sanitation activities. Hybrid trials that blend efficacy and effectiveness (including randomized trials) and implementation research studies will be necessary for implementing simultaneous process evaluations that provide context for clinical findings and inform future guidelines. Perhaps the largest undertaking on the research agenda will be defining, testing, and validating STH transmission breakpoints. Meanwhile, ministries of health will be challenged by partnering with researchers to facilitate the intensified trials in select areas while continuing STH control policies broadly.

In light of these research needs, the Bill & Melinda Gates Foundation recently launched the DeWorm3 Project in partnership with the Natural History Museum (NHM) in London. The purpose of DeWorm3 is to test the feasibility of interrupting transmission of STHs by leveraging existing LF elimination efforts. The project will require cluster randomized trials of a number of intensified STH interventions, including community-wide MDA, cost-effectiveness analyses, qualitative studies of compliance and stakeholder satisfaction, mathematical modeling exercises, drug resistance monitoring, and a number of other research initiatives that explore the unique opportunity to innovatively transition from STH control to elimination strategies.

The prospect of leveraging LF platforms to benefit STH programs in co-endemic areas has been promoted since the initial establishment of the Global Alliance to Eliminate Lymphatic Filariasis (GAELF) in 2000 [15]. As an increasing number of LF programs transition to post-MDA surveillance, the opportunity to leverage these valuable platforms to sustain the gains observed in STH co-endemic areas has never been more clear, nor more timely. This is a moment of enormous opportunity for programs, policy makers, and communities to come together to eliminate the public health risks associated with STH infection.
